# Species-dependent extracranial manifestations of a brain seeking breast cancer cell line

**DOI:** 10.1371/journal.pone.0208340

**Published:** 2018-12-10

**Authors:** Valerie De Meulenaere, Sara Neyt, Bert Vandeghinste, Pieter Mollet, Olivier De Wever, Elke Decrock, Luc Leybaert, Ingeborg Goethals, Christian Vanhove, Benedicte Descamps, Karel Deblaere

**Affiliations:** 1 Department of Radiology, Ghent University Hospital, Ghent, Belgium; 2 MOLECUBES NV, Ghent, Belgium; 3 Department of Experimental Cancer Research, Ghent University, Ghent, Belgium; 4 Department of Basic Medical Sciences, Ghent University, Ghent, Belgium; 5 Department of Nuclear Medicine, Ghent University Hospital, Ghent, Belgium; 6 IBiTech—Medisip—Infinity lab, Ghent University, Ghent, Belgium; University of South Alabama Mitchell Cancer Institute, UNITED STATES

## Abstract

**Purpose:**

Metastatic brain tumors pose a severe problem in the treatment of patients with breast carcinoma. Preclinical models have been shown to play an important role in unraveling the underlying mechanisms behind the metastatic process and evaluation of new therapeutic approaches. As the size of the rat brain allows improved in vivo imaging, we attempted to establish a rat model for breast cancer brain metastasis that allows follow-up by 7 tesla (7T) preclinical Magnetic Resonance Imaging (MRI).

**Procedures:**

Green fluorescent protein-transduced (eGFP) MDA-MB-231br breast cancer cells were labeled with micron-sized particles of iron oxide (MPIOs) and intracardially injected in the left ventricle of female nude rats and mice. 7T preclinical MRI was performed to show the initial distribution of MPIO-labeled cancer cells and to visualize metastasis in the brain. Occurrence of potential metastasis outside the brain was evaluated by 2-deoxy-2-[^18^F]fluoro-D-glucose ([^18^F]FDG) positron emission tomography (PET)/computed tomography (CT) and potential bone lesions were assessed using [^18^F]sodium fluoride ([^18^F]NaF) PET/CT.

**Results:**

The first signs of brain metastasis development were visible as hyperintensities on T2-weighted (T2w) MR images acquired 3 weeks after intracardiac injection in rats and mice. Early formation of unexpected bone metastasis in rats was clinically observed and assessed using PET/CT. Almost no bone metastasis development was observed in mice after PET/CT evaluation.

**Conclusions:**

Our results suggest that the metastatic propensity of the MDA-MB-231br/eGFP cancer cell line outside the brain is species-dependent. Because of early and abundant formation of bone metastasis with the MDA-MB-231br/eGFP cancer cell line, this rat model is currently not suitable for investigating brain metastasis as a single disease model nor for evaluation of novel brain metastasis treatment strategies.

## Introduction

Tumor metastasis is the leading cause of cancer death worldwide. Breast carcinomas frequently metastasize to the brain with brain metastasis occurring in up to 10–15% of breast cancer patients with metastatic disease [1]. Standard medical treatment of brain metastases (chemotherapy, whole brain radiotherapy and surgery) is often ineffective due to a suggested low permeability of the blood-brain barrier (BBB) to therapeutic agents, high sensitivity of healthy brain tissue for radiation or the presence of metastatic tumors near critical structures in the brain [2].

Cancer metastasis formation entails a strong component of tumor-host interactions and usually requires several essential steps: intravasation into bloodstream, arrest at vascular branch points, extravasation, persistent close contact to microvessels and perivascular growth by angiogenesis or vessel cooption [3, 4]. Moreover, Kienast et al. observed that a perpetuation of a strict perivascular position, with physical contact between the extravasated human lung and melanoma cancer cell and the abluminal endothelial cell of a brain capillary, is essential for successful metastasis growth [3].

Various preclinical animal models to study brain metastasis were summarized by Eichler et al. [4]. Most of the currently available models bypass the initial steps of the metastatic process due to implantation of the tumor in the brain. Orthotopic implantation of primary tumors that metastasize spontaneously often leads to systemic disease and death of the animals before the brain can be reliably examined [4]. Ectopic inoculation of cancer cells into the bloodstream via tail vein injection results in dissemination of cancer cells primarily to the lungs with further metastasis to the central nerve system and leads almost inevitably to lung metastases. To avoid passage of cancer cells into the pulmonary system, intracardiac injection into the left ventricle of the heart can be an alternative. This type of injection leads to a systemic distribution of the cancer cells to most organs [5]. The initial delivery of cancer cells to an organ after intracardiac injection depends on the cardiac output to the organ with about 2.8% of the cancer cells being delivered to the rat brain [6, 7]. Ideally, the experimental brain metastasis model should closely mimic human disease so therefore intracardiac injection is the most preferable method to induce metastasis in the brain.

Yoneda et al. developed a brain-seeking clone of the MDA-MB-231 cell line derived from a triple negative breast cancer patient (MDA-MB-231br) by performing repeated sequential passages of metastatic cancer cells obtained from brain metastases in nude mice [8]. This MDA-MB-231br cancer cell line was described to exclusively disseminate to the mouse brain.

Several in vivo imaging modalities can be used for the detection of brain metastases. Up to now, Magnetic Resonance Imaging (MRI) is the gold standard for the clinical evaluation of brain metastases and is well suited for longitudinal follow-up. Gadolinium (Gd)-based contrast-enhanced MRI is widely considered as the most accurate imaging tool for diagnosis of brain tumors [9]. The advantage of MRI is the excellent soft tissue contrast and high in vivo spatial resolution resulting in anatomical detail. In order to detect specific cells, a dedicated MRI contrast agent is needed. Micron-sized particles of iron oxide (MPIOs) can be used as a T2 contrast agent as they create small hypointense signal voids on conventional T2-weighted (T2w) and T2*-weighted (T2*w) images [10]. Iron oxide particles are widely used for cell labeling since they are biocompatible and therefore can be recycled in the normal iron metabolism [11]. In vitro labeling of cancer cells shows efficient internalization of MPIOs [10]. Many research groups have used these particles in several animal models as they allow tracking from the single-cell stage until the appearance of overt brain metastases [12].

Rats are an ideal model for imaging in part due to their more translational nature and closer physiology to humans compared to mouse [13]. The greater size of rats also provides advantages for the intracardiac injections of the cancer cells as a bigger heart consequently leads to easier intracardiac injections with fewer errors, especially in combination with US guidance to target the left ventricle. Another advantage is that the larger size of a rat brain offers better relative spatial resolution and therefore allows improved imaging compared to a mouse model for brain metastases [13]. Moreover, clinical deterioration is to be expected to a lesser extent in function of total brain volume compared to mice.

Since preclinical models can play an important role for the study of cancer and the development of new therapeutic approaches, we attempted to establish a clinically relevant rat model for brain metastases with MPIO-labeled MDA-MB-231br/eGFP cancer cells.

## Materials and methods

### Cell culture and MPIO labeling

The development of the MDA-MB-231br/eGFP breast cancer cell line has previously been described [8, 14]. MDA-MB-231br/eGFP cancer cells were maintained in Dulbecco’s modified Eagle’s medium (DMEM) supplemented with 10% fetal calf serum (FCS), 1% penicillin-streptomycin antibiotics, 0.0005% fungizone, 1% pyruvate and 1mg/ml geneticin at 37°C and 10% CO_2_. Labeling of these cancer cells was done by adding 100 MPIOs/cell (MPIO; 1 μm, Dynabeads MyOne, Invitrogen, Oslo, Norway) to the cell culture medium for 24 hours [10]. Subsequently, the cells were washed extensively with phosphate buffered saline (PBS) to remove unincorporated MPIOs. MPIO-labeled cancer cells were visualized via phase contrast microscopy (Leica DMI3000 microscope, Leica, Rijswijk, The Netherlands). A trypan blue exclusion assay was assessed to determine the viability of the cancer cells.

### Animal models

This study protocol was approved by the Ghent University ethics committee for animal experiments (ECD 14/18 and ECD 17/19). All animals were kept and handled according to the European guidelines and housed under environmentally controlled conditions (12h normal light/dark cycles, 20°C– 24°C and 40–70% relative humidity) with food and water ad libitum. Animals were fasted overnight before [^18^F]FDG PET scans were performed.

Female nude rats (n = 13, 5-weeks old, Crl:NIH-*Foxn1*^*rnu*^, Charles River) and female nude mice (n = 5, 5-weeks old, BALB/cOlaHsd-*Foxn1*^*nu*^, Envigo) were intracardially injected with MPIO-labeled MDA-MB-231br/eGFP cancer cells (100.000 and 50.000 cells, respectively) using US guidance to target the left ventricle. To examine the effect of MPIO-labeling on metastasis development, additional female nude rats (n = 8, 5-weeks old, Crl:NIH-*Foxn1*^*rnu*^, Charles River) were intracardially injected with 100.000 cancer cells without MPIO-label.

The procedure for intracardiac injection has previously been described for mice and is similar for rats [15]. Rats were anesthetized using 1.5% isoflurane gas mixed with oxygen administered at a flow rate of 0.2 l/min and placed supine with all four limbs fixated on the heated stage. Ultrasonographic gel was applied and an US probe (FUJIFILM VisualSonics, Vevo 2100, Toronto, Canada) was used to find the left ventricle of the heart. A syringe, secured into a holder, containing a 400 μl suspension of MPIO-labeled or unlabeled cancer cells was injected slowly after visual confirmation of the needle (3/4-inch-long 27-gauge) in the left ventricle of the heart. After intracardiac injection, rats and mice were examined weekly by MRI during 6 weeks to monitor brain metastasis development. Taking the human endpoints into account, animals were euthanized when paralysis, deterioration, weakness or weight loss (>20%) was observed.

### Multi-modal imaging

Although the cell line was transduced with eGFP and therefore allowed detection of cancer cells using fluorescence imaging, other advanced imaging techniques such as MRI, positron emission tomography (PET) and computed tomography (CT) were used for follow-up of metastasis development.

#### MRI for visualization of brain metastases

MRI was performed on a 7T system (PharmaScan 70/16, Bruker, Ettlingen, Germany) to show the initial distribution of MPIO-labeled cancer cells and to visualize metastasis development in the brain (Table 1).

**Table 1:** Overview of scans acquired at several time points post-injection (PI).

GROUP	n =	CELL LINE	LABEL	MRI FOLLOW-UP			[^18^F]FDG PET/HIGH RESOLUTION CT	[^18^F]NaF PET/CT	
				T2*	T2	T1+Gd		Early	Late
RAT_MPIO	13	MDA-MB-231br/eGFP	MPIO	24h PI	Week 3, 4, 5 and 6 PI	Week 3, 4, 5 and 6 PI	4–5 weeks PI	2 weeks PI	4–5 weeks PI
RAT_NOMPIO	8	MDA-MB-231br/eGFP	/	/	Week 3, 4, 5 and 6 PI	Week 3, 4, 5 and 6 PI	4–5 weeks PI	2 weeks PI	4–5 weeks PI
MOUSE_MPIO	5	MDA-MB-231br/eGFP	MPIO	24h PI	Week 3, 4, 5 and 6 PI	/	4–5 weeks PI	2 weeks PI	4–5 weeks PI

The animals were anesthetized with isoflurane and O_2_ and through a nose cone fixed on the Bruker rat restrainer. A heating pad was placed beneath each animal to maintain body temperature at 37°C before they were placed inside the magnet.

Initial cell arrest was assessed 24 hours post-injection (PI) with 7T MRI of the brain using T2*w images (GRE FLASH, 120μm isotropic resolution, TR/TE 50/14ms) susceptible to the local change in magnetic field caused by MPIOs. With the use of T2w MR images (SE RARE, 109μm in-plane resolution, TR/TE 6346/37ms for rats–TR/TE 3700/37ms for mice, slice thickness: 0.6 mm) parenchymal metastases were visualized 3, 4, 5 and 6 weeks PI. Contrast-enhanced T1-weighted (T1w) sequences (SE RARE, 117μm in-plane resolution, TR/TE 1382/9.7ms; 2 mmol/kg, Dotarem, Guerbet) were acquired in rats to demonstrate the leaky BBB present in some brain metastases. Gd-based contrast agent was intravenously injected into a tail vein of the rats. To reduce the risk of death due to prolonged anesthesia, no contrast-enhanced T1w sequences were run for mice.

#### PET/CT for visualization of extracranial metastasis

Static whole-body [^18^F]FDG PET/CT (20 min acquisition time for rats and 10 min acquisition time for mice, β-CUBE, MOLECUBES NV, Ghent, Belgium) was assessed to evaluate potential metastasis development outside the brain and static whole-body [^18^F]NaF PET/CT (15 min acquisition time for rats and 10 min acquisition time for mice, β-CUBE, MOLECUBES NV, Ghent, Belgium) was performed to determine possible bone lesions. [^18^F]FDG PET/CT scans were acquired 4–5 weeks post-injection and [^18^F]NaF PET/CT scans were obtained at 2 different time points: 2 (early) and 4–5 (late) weeks PI (Table 1). Rats and mice were anesthetized with isoflurane and O_2_ for the duration of the PET/CT acquisitions. Polyethylene tubing was placed in the lateral tail vein to allow intravenous injection of a radioactive tracer (10 MBq for rats and mice). After tracer uptake (30 minutes for [^18^F]FDG and [^18^F]NaF), the animals were imaged with their body temperature maintained at 37°C using a heated bed.

PET data were reconstructed using an OSEM algorithm with 50 iterations and a reconstructed voxel size of 400 μm. A high-resolution CT acquisition (X-CUBE, MOLECUBES, Ghent, Belgium) was done for anatomical correlation and detection of possible bone metastasis.

PET images, both as single images and in combination with the automatically co-registered CT scan, were interpreted by an experienced clinical radiologist (KD) and a nuclear medicine physician (IG) for the presence of bone lesions.

#### High-resolution CT for visualization of bone metastasis

For anatomical correlation and detection of possible bone metastases full-body spiral high-resolution CT acquisitions (7 minutes acquisition for rats and 3 minutes acquisition for mice, X-CUBE, MOLECUBES NV, Ghent, Belgium) with 460 μA tube current and 50 kVp tube voltage were performed. The full body spiral scans were reconstructed using an iterative algorithm (ISRA) with a voxel size of 200 μm. Different subvolumes were reconstructed into a 1400x1400x4000 matrix with 50 μm voxel size using an FDK-based algorithm.

### Histological confirmation

At the end of the experiment or when the human endpoints were reached, animals were euthanized by an intravenous injection of pentobarbital (120 mg/kg). For a selection of animals the brains were isolated, immersed for 24 hours in 4% paraformaldehyde and embedded in paraffin. Then, cerebrum and cerebellum were partly sectioned in 5μm slices and stained with Hematoxylin and Eosin (H&E) for histological confirmation of brain metastasis development.

### Image analysis

MPIO-induced hypointensities were analyzed on T2*w MR images using OsiriX software (OsiriX v.5.8.1). A single observer counted the number of hypointensities in a standardized manner using minimal intensity projections of T2*w images with a slice thickness of 0.42 mm starting from olfactory bulb to midbrain, 20 slices for mice and 25 slices for rats (slice thickness: 0.12 mm). Total volume of brain metastases and number of brain metastases were assessed on T2w MR images. Brain metastases volumes were measured by manually outlining hyperintense regions on individual slices of T2w MR images using OsiriX software. The obtained tumor areas were then multiplied by the slice thickness (0.6 mm) to calculate the volume of each brain metastasis. Bone metastasis development was analyzed on high-resolution CT images acquired 4–5 weeks after intracardiac injection through counting the number of metastasis affected bones.

### Statistical analysis

Statistical analyses were done using SPSS software (SPSS Statistics 25). For the outcomes ‘*number of brain metastases*’ and ‘*number of metastasis affected bones*’ a generalized linear mixed model with a negative binomial distribution was applied. For the outcome ‘number of affected animals’ a generalized linear mixed model with a binary logistic regression was used. All other outcomes were statistically analyzed using a linear mixed model. ‘*Series*’ were defined as a random effect in all analyses. Scatterplots were used to describe the relation between ‘*number of hypointensities*’ and ‘*number of brain metastases*’. Kaplan-Meier curves were generated to estimate time between intracardiac injection and clinical symptoms and were compared by species using the log-rank test. P values were considered significant if they were less than 0.05. Values are presented as mean ± SE.

## Results

### Development of animal models for breast cancer brain metastasis and follow-up by multimodal imaging

**Fig 1:**
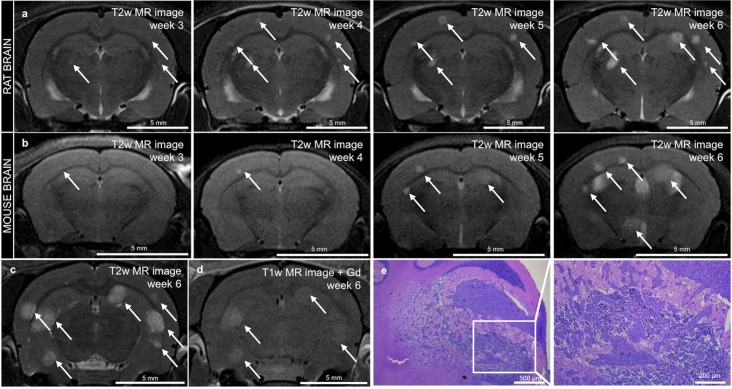
MR imaging and histological confirmation of brain metastasis development. a-b. In vivo serial T2w MRI scans in the same rat (a) and mouse (b) showing metastasis development as soon as 3 weeks after intracardiac injection (metastases are indicated with an arrow). c-d. T2w MR image (c) and T1w MR image after Gd enhancement (d) both acquired in a rat brain 6 weeks after intracardiac injection (metastases are indicated with an arrow). e. Histological confirmation of metastasis by H&E staining on paraffin-embedded slices in the cerebellum of a rat.

US guidance allowed successful cancer cell injection into the left ventricle of the heart in all female nude rats and mice (5-weeks old). T2*w MR images showed the initial distribution of MPIO-labeled cancer cells as hypointensities in the rat and mouse brain and were used as a control step for proper injection of the cancer cells. For brain metastasis follow-up, T2w and contrast-enhanced T1w MR images were acquired. Three weeks after intracardiac injection, the first signs of metastasis development were visible as hyperintensities on T2w MR images of rats (Fig 1A) and mice (Fig 1B). T1w images showed a heterogeneous enhancement, which was not related to metastasis size (Fig 1C and 1D). Consequently, T1w images were not used for further analysis of brain metastases numbers and volumes. H&E staining performed on paraffin-embedded slices of cerebrum and cerebellum at the end of the experiment confirmed metastasis development in the brain (Fig 1E).

#### Relation between hypointense signal voids and brain metastases

**Fig 2:**
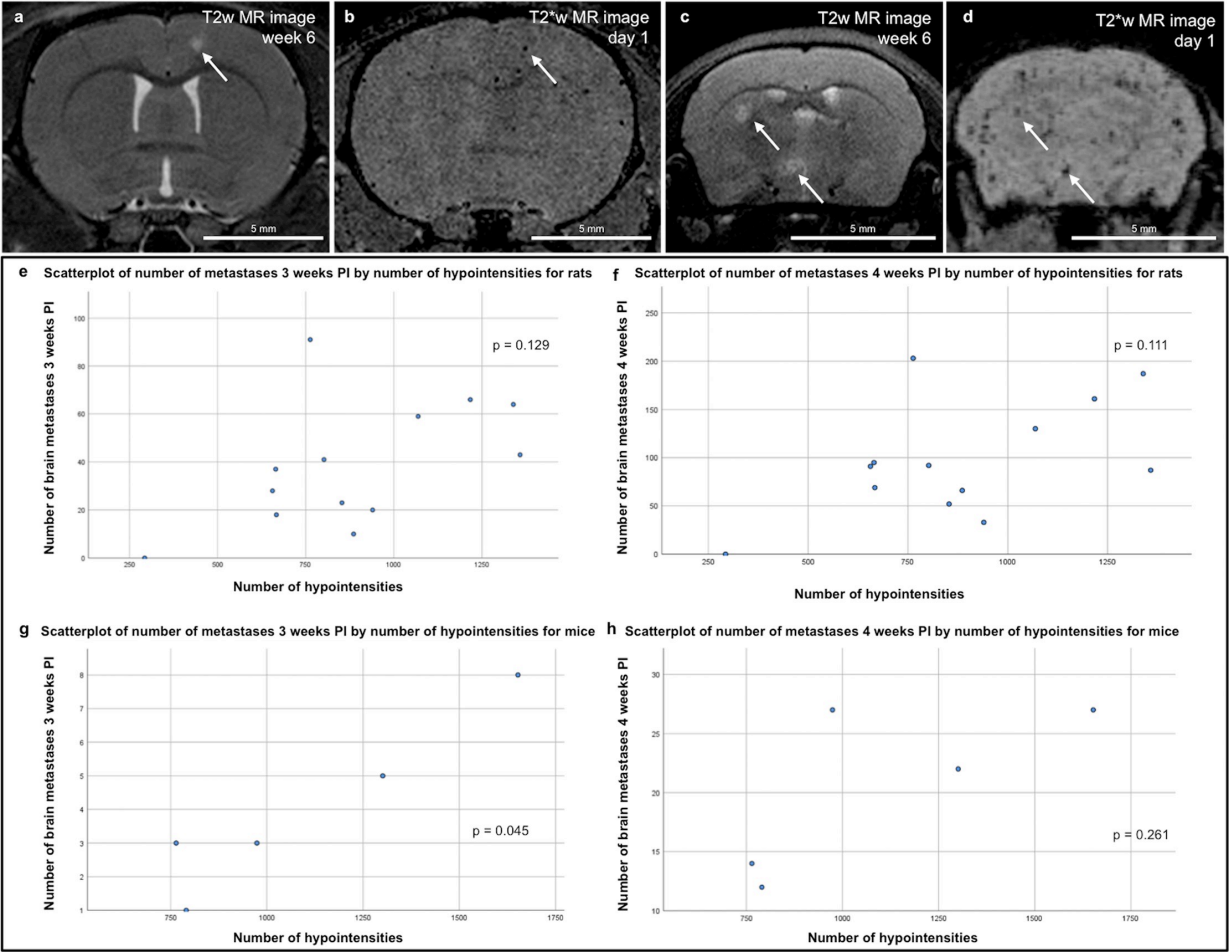
Correlation of brain metastasis and hypointensities. a-b. Rat brain metastasis visible on a T2w image (a) could be correlated to a corresponding hypointense signal void on a T2*w image (b) as indicated with an arrow. c-d. Mouse brain metastases visible on a T2w image (c) could be correlated to corresponding signal voids on a T2*w image (d) as indicated with an arrow. e-h. Scatterplots describe the relation between ‘number of hypointensities’ and ‘number of brain metastases’ for rats (n = 13) injected with MPIO-labeled cancer cells 3 and 4 weeks PI (e and f, respectively) and mice (n = 5) injected with MPIO-cancer cells 3 and 4 weeks PI (g and h, respectively).

Rats injected with 100.000 MPIO-labeled cancer cells showed 885 ± 303 hypointense signal voids on T2*w images, while mice injected with 50.000 cancer cells showed 1096 ± 377 hypointense signal voids. For a subset of brain metastases visible on T2w images correlation to their corresponding hypointensities on T2*w images was confirmed (Fig 2A–2D). The relation between number of hypointensities and number of brain metastases was described by scatterplots (Fig 2E–2H). For rats, no significant correlation was found between number of hypointensities measured 24 hours PI and number of brain metastases measured 3 and 4 weeks PI (p = 0.129 and p = 0.111, respectively) (Fig 2E and Fig 2F, respectively). For mice, a significant correlation between number of hypointensities measured 24 hours PI and number of brain metastases measured 3 weeks PI was found (p = 0.045) (Fig 2G). This correlation was no longer detectable 4 weeks PI (p = 0.261) (Fig 2H).

#### Analysis of brain metastases

**Fig 3:**
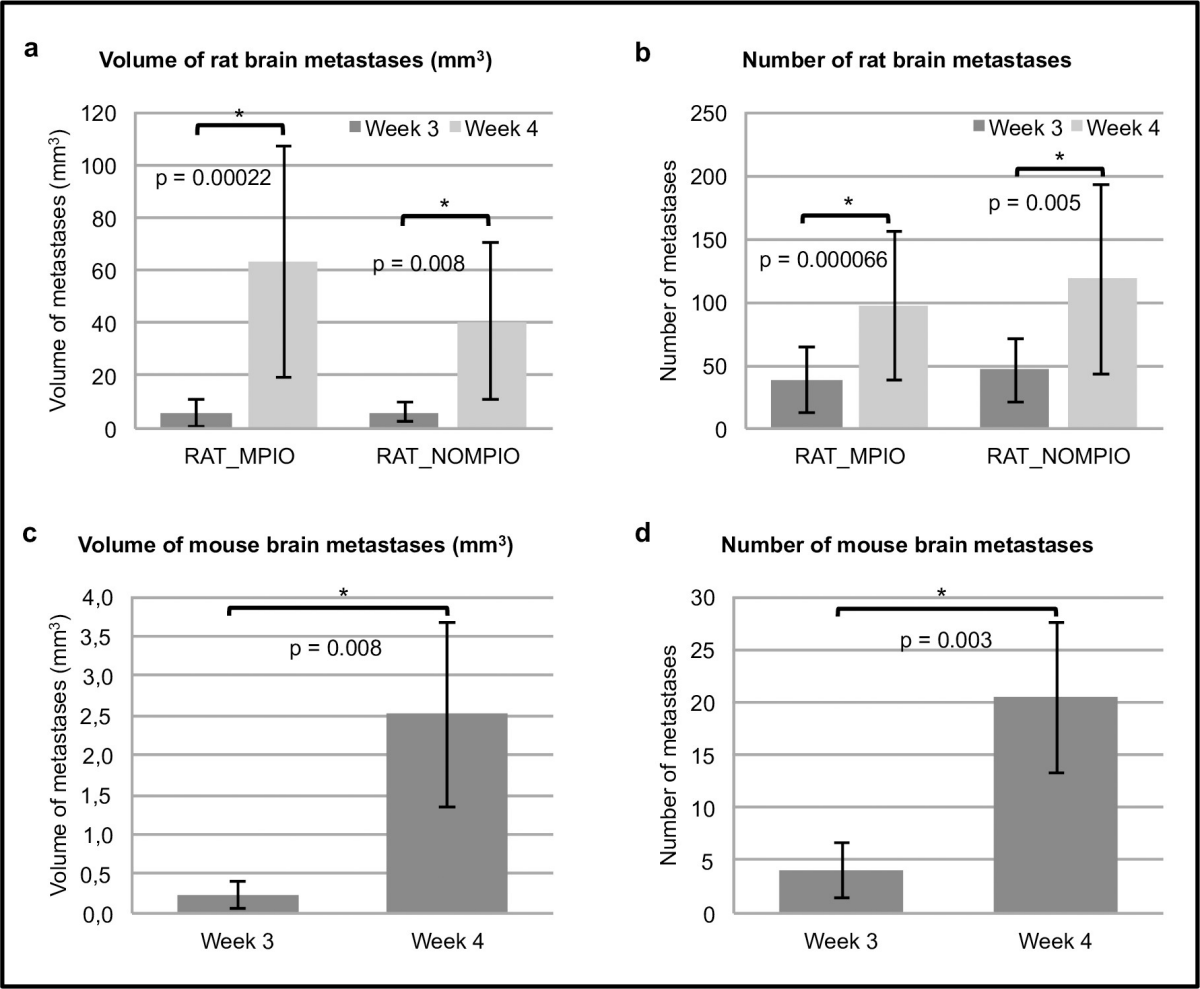
Brain metastases analyses. a. Total volume of brain metastases (mm^3^) of rats injected with MPIO-labeled (n = 13) or unlabeled (n = 8) cancer cells; data acquired 3 and 4 weeks post-injection. b. Total number of brain metastases of rats injected with MPIO-labeled (n = 13) or unlabeled (n = 8) cancer cells; data acquired 3 and 4 weeks post-injection. c. Total volume of brain metastases (mm^3^) of mice (n = 5) injected with MPIO-cancer cells; data acquired 3 and 4 weeks post-injection. d. Total number of brain metastases of mice (n = 5) injected with MPIO-labeled cancer cells; data acquired 3 and 4 weeks post-injection. a-d. For all 3 groups, a significant difference was found between number of metastases measured 3 and 4 weeks PI and also between volume of metastases measured 3 and 4 weeks PI.

Results of brain metastases analysis are shown in Fig 3 for rats injected with MPIO-labeled or unlabeled cancer cells and for mice injected with MPIO-labeled cancer cells. 76% of all rats (16/21) and 20% of all mice (1/5) were suffering from their metastasis development and were euthanized between 4 to 6 weeks PI. Therefore, statistical analysis was performed on data acquired 3 and 4 weeks after intracardiac injection of the cancer cells, when all animals were still alive.

#### Analysis of extracranial metastases

**Fig 4:**
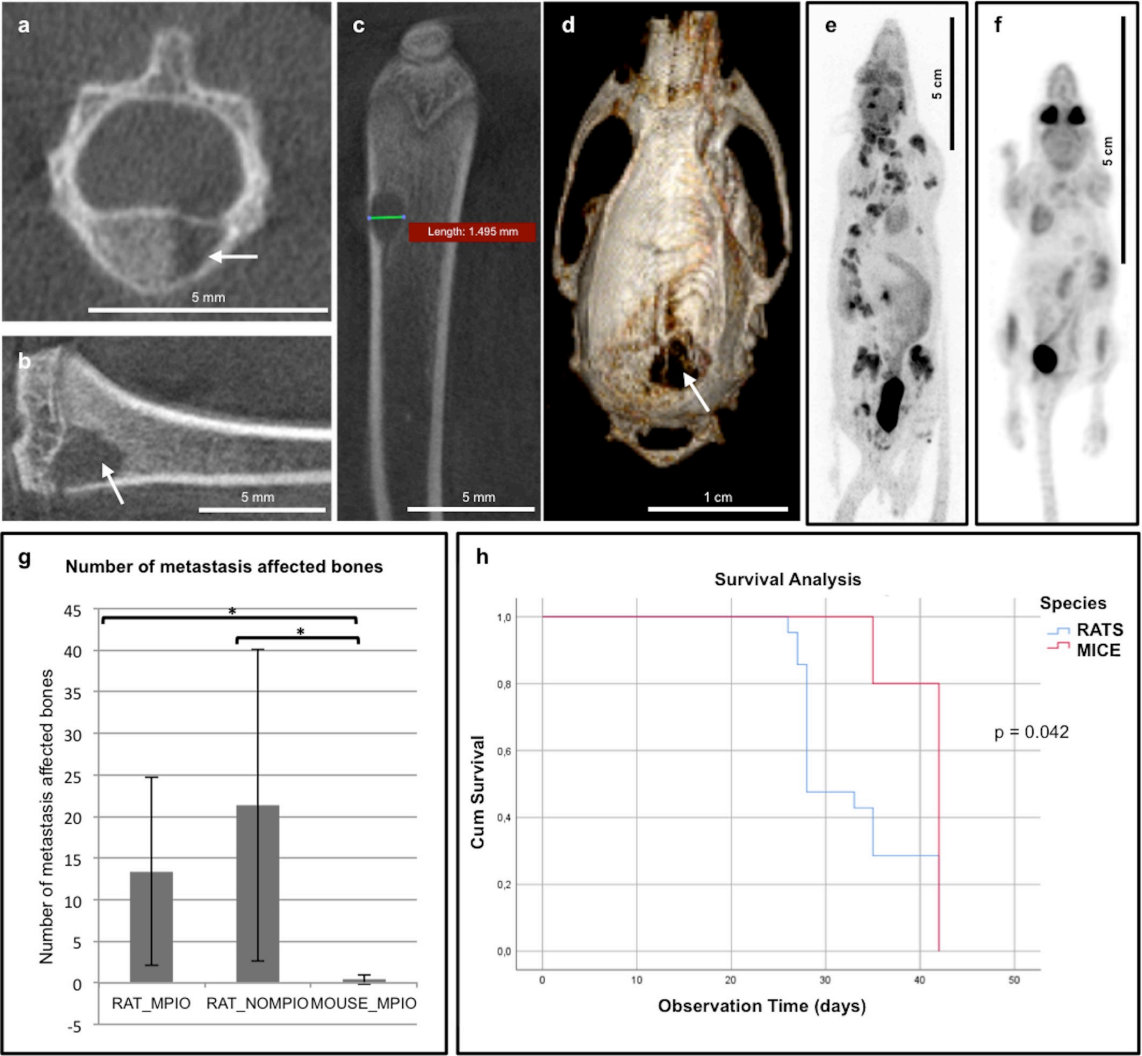
Bone metastases analysis. a-d. High-resolution μCT images acquired on X-CUBE (recon: FDK—50 μm). Evaluation showed metastasis throughout the skeleton in 81% (17/21) of the rats. Examples of lytic bone lesions detected in vertebra (a), tibia (b), femur (c) and skull (d) are indicated with an arrow. e-f. Static full-body [^18^F]FDG PET image showing metastasis in the skeleton of rat (e) and no metastasis in the skeleton of mouse (f). g. Number of metastasis affected bones showing a significant difference between rats and mice. h. Kaplan Meier curves and log-rank test demonstrate a significant difference in clinical symptoms free survival times between rats and mice.

After intracardiac injection of MPIO-labeled cancer cells, 11 out of 13 rats developed bone metastases with 13 ± 11 affected bones per animal. These bone metastases were mainly osteolytic, characterized by pathological destruction of the bone, and thus clearly visible on high-resolution CT images acquired 4–5 weeks PI (Fig 4A–4D). When unlabeled cancer cells were injected 6 out of 8 rats developed bone metastases with 21 ± 19 affected bones per animal. In mice, one affected bone was found in 2 out of 5 animals with 0.4 ± 0.55 affected bones per animal.

### Influence of MPIO-labeling on metastasis

The possible influence of MPIO-labeling on cancer metastasis to brain and bone was assessed using preclinical brain MRI and whole-body PET/CT. Evaluation of brain metastases showed no significant differences between the number of brain metastases and the brain metastases volume whether the rats were injected with MPIO-labeled or unlabeled cancer cells (for number of brain metastases: p = 0.603 and p = 0.64, respectively—for brain metastases volumes: p = 0.951 and p = 0.218, respectively) (Fig 3A and 3B). Moreover, bone metastasis development and number of metastasis affected bones did also not significantly differ among rats whether they were injected with MPIO-labeled or unlabeled cancer cells (p = 0.603 and p = 0.418, respectively) (Fig 4G). These findings demonstrate that MPIO-labeling had no influence on metastasis development in brain or bone. Furthermore, tumors had an identical appearance after injection of MPIO-labeled or unlabeled cancer cells, showing that the overall function of cancer cells was also not affected by MPIO-labeling.

### Influence of species on metastasis

Brain metastasis development was visible in all rats and mice. Different brain volumes and cancer cell dissemination (i.e. number of hypointensities on T2*w MR images) made a comparison between rats and mice regarding analysis of brain metastases size difficult. Although mice were injected with 50.000 cancer cells, compared to 100.000 cancer cells in rats, a higher number of hypointensities 24 hours PI was observed and caused a lower number of brain metastases 3 and 4 weeks PI.

For rats, there is a clear trend (p = 0.097) towards higher odds (OR = 6.25 and 95% CI {0.7; 58.82}) for bone metastasis development compared to mice. As only one affected bone was found in 2 out of 5 mice, the number of metastasis affected bones in mice was 0.4 ± 0.55, which is significantly lower (p = 0.003) compared to rats (Fig 4E–4G).

Kaplan Meier curves and log-rank test demonstrated a significant difference in time between intracardiac injection and clinical symptoms between rats and mice (p = 0.042) with an estimated median clinical symptoms free survival time of 28 ± 1.5 days for rats and 42 ± 0 days for mice (Fig 4H).

### Timing and tracer choice for preclinical evaluation of whole body bone metastases

**Fig 5:**
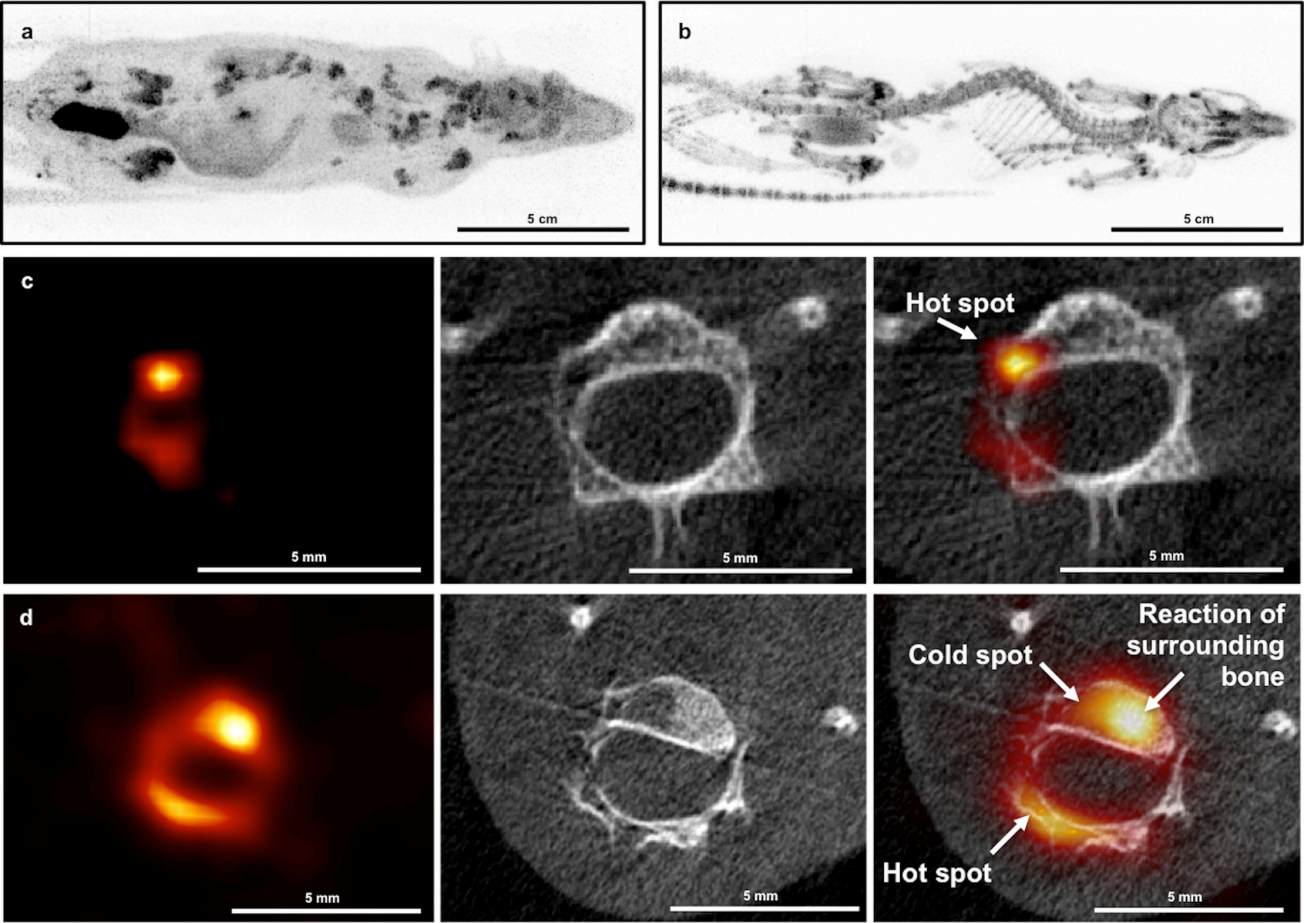
[^18^F]FDG and [^18^F]NaF PET imaging in rats. a. Static whole-body rat [^18^F]FDG PET image. b. Static whole-body rat [^18^F]NaF PET image. c. PET-CT image of rat vertebra; [^18^F]FDG PET image anatomically correlated with high resolution CT (recon: FDK—50 μm). This lytic lesion is clearly visible on the [^18^F]FDG PET images as a hypermetabolic lesion (‘*hot spot*’) d. PET-CT image of rat vertebra; [^18^F]NaF PET image anatomically correlated with high resolution CT (recon: FDK—50 μm). The bone lesions have a mixed appearance: both ‘*hot spots*’ and ‘*cold spots*’ are visualized in the lytic CT lesions, probably depending on the stage of destruction and reaction of the surrounding bone.

Two weeks following intracardiac injection of the cancer cells, no bone lesions were observed on the [^18^F]NaF PET images and the corresponding CT scan in both rats and mice. In a later stage, 4–5 weeks PI, osteolytic bone metastases were visualized on the high-resolution CT images. These osteolytic bone metastases were clearly visible on the [^18^F]FDG PET images as hypermetabolic lesions (‘*hot spots*’) (Fig 5A and 5C). On the [^18^F]NaF PET images these lesions had a mixed appearance: both ‘*hot spots*’ and ‘*cold spots*’ were visualized in the osteolytic CT lesions (Fig 5B and 5D).

Asymmetric tracer uptake was fundamental for diagnosis of bone metastases on PET images. The heterogeneous appearance on [^18^F]NaF PET made diagnosis of all bone metastases in the animals nearly impossible, especially without the use of simultaneously acquired CT scan. Moreover, high-resolution CT acquired in combination with the [^18^F]FDG PET and [^18^F]NaF PET images was essential for diagnosis of bone metastases in the vicinity of the growth plates present in these young animals.

## Discussion

We evaluated the behavior of the human MDA-MB-231br/eGFP breast cancer cell line in rats using advanced imaging techniques such as MRI, PET and CT in order to develop a rat model for breast cancer brain metastasis that will allow for future evaluation of the effectiveness of new therapeutic approaches. The most preferable method to induce metastasis in the brain is intracardiac injection of brain seeking cancer cells, in this case MDA-MB-231br/eGFP cancer cells. Intracardiac injections guided by ultrasound were easy to perform and showed a 100% success rate without adverse events. They also resulted in a more reliable distribution and reproducible delivery of cancer cells to the brain, compared to uncontrolled intracardiac injection [15].

Up to now, MRI is the gold standard for clinical evaluation of brain metastases. For this study, preclinical MRI was used because of its noninvasive nature, diverse set of image contrast possibilities, high spatial resolution (up to 50 μm at 7T) and high signal-to-noise ratio. MRI allows longitudinal follow-up and is an excellent tool for the study of tumor growth and therapy response over time [6]. Initial cancer cell arrest in the brain was assessed using T2*w images susceptible to the local change in magnetic field caused by MPIOs. When the MDA-MB-231br/eGFP cancer cells are labeled with MPIOs, tracking from the cellular stage until the appearance of overt metastases is possible [12]. Sundstrøm et al. suggested that quantitative tracking of melanoma cells labeled with iron oxide nanoparticles could improve the predictive power of an experimental brain metastasis model by controlling variation due to the heterogeneous nature of metastatic melanoma and inherent technical limitations [16]. Since variation in dissemination could affect experimental reproducibility, selection of animals with comparable brain dissemination (i.e. number of hypointensities on T2*w MR images) can be crucial [16, 17]. Our procedure of counting hypointensities was not without limitations. T2*w images of the hindbrain were occasionally of minor quality compared to forebrain and midbrain, therefore, hypointensities were only manually counted from olfactory bulb to midbrain. Automated counting of hypointensities was considered but, as clustering made it difficult to differentiate hypointense signal voids as single or multiple MPIO-labeled cancer cells, withheld. For our rat model for brain metastasis no significant correlation between cancer cell dissemination and number of brain metastases was found, even when the number of injected cancer cells was repeatedly the same, which makes selection based on initial brain dissemination not useful. Larger differences in number of injected cells could possibly lead to a more distinct correlation [12]. In our study, all rats were injected with 100.000 cancer cells, this high cell density could have caused aggregation of discrete signal voids and therefore difficulties in counting the hypointensities leading to an underestimation of cancer cell load in the brain as also described by Heyn et al. [12]. Since mice were injected with a lower number of cancer cells (i.e. 50.000 cancer cells), less aggregation of discrete signal voids was demonstrated. On the other hand, several physiological factors such as cardiac output determine the distribution of cancer cell dissemination in the brain. While for rats about 2.8% of the cancer cells will be delivered to the brain after intracardiac injection, mice show a cardiac output to the brain of about 3.5–9.5% [6, 7]. This might also contribute to the higher number of hypointensities measured for mice compared to rats.

With the use of T2w MR images, the first parenchymal metastases were observed 3 weeks after intracardiac injection. At 6 weeks PI, some of these metastases were characterized by a leaky BBB as demonstrated by Gd enhancement on T1w MR images. As such, analysis of the metastatic lesions was preferably done on T2w images given the complicated relation between size of the lesions and permeability for the Gd contrast agent [18–20]. Larger metastasis size was more likely to show Gd enhancement, but this was certainly not the case for all larger lesions (Fig 1C and 1D). An important limitation concerning assessment of metastatic volume on T2w MR images is inclusion of tumor-associated edema leading to an overestimation of the actual tumor size [19]. On the other hand, using T1w MRI after Gd administration for the assessment of total tumor burden in each brain would have most certainly led to an underestimation of total metastases volume as numerous smaller lesions demonstrated no enhancement on these T1w images. Therefore, we opted to use T2w MRI to assess number and volume of brain metastases.

Early formation of metastases outside the brain, especially in bone, was clinically observed. These bone lesions caused severe clinical symptoms resulting in early euthanasia of some rats (Fig 4H). This prompted us to assess bone metastasis formation. Evaluation of bone metastases is generally done via [^99m^Tc]methylene diphosphonate ([^99m^Tc]MDP) bone scintigraphy, hereby detecting increased osteoblastic activity characterized by excessive new but disorganized bone formation [21]. Additionally, [^18^F]FDG and [^18^F]NaF can be used to evaluate bone metastasis with PET [22]. PET enables non-invasive in vivo monitoring of the metabolic process and can provide relevant information on tumor metabolism [22–24]. Compared to [^99m^Tc]MDP bone scintigraphy, PET has a higher spatial resolution as well as a higher specificity and sensitivity for detection of bone metastases [24]. [^18^F]FDG PET visualizes tumor cells with increased metabolic activity within the bone marrow. As osteolytic lesions show a high glycolytic rate, [^18^F]FDG PET is the preferable method for detection of this type of lesions [21, 25]. Osteoblastic lesions, however, are preferably detected by [^18^F]NaF PET as the mechanism of uptake of [^18^F]NaF is related to local blood flow and osteoblastic activity [26]. Static whole-body [^18^F]FDG PET/CT scans were performed 4–5 weeks post-injection and [^18^F]NaF PET/CT scans were obtained at 2 different time points: 2 (early) and 4–5 (late) weeks PI. Two weeks following intracardiac injection, no bone lesions were observed on the [^18^F]NaF PET images and the corresponding CT scan, which was probably due to the small size of the lesions. In a later stage, 4–5 weeks following the intracardiac injection, mainly lytic bone metastases were visualized on the high-resolution CT images. These results are in line with the observation that the original MDA-MB-231 cell line also mostly develops lytic bone metastases [27]. Hence, [^18^F]FDG PET is the preferable detection method in our model [8]. In addition, high-resolution CT provides morphological information and was essential for diagnosis of bone lesions when acquired in combination with the [^18^F]FDG PET images. High-resolution CT was especially crucial for diagnosis of bone lesions in the vicinity of growth plates present in these young rats, since both bone lesions as well as growth plates were visible as hypermetabolic regions (hot spots) on the [^18^F]FDG PET images.

In order to optimize the model, the possible influence of MPIO-labeling on cancer metastasis to brain and bone was evaluated in rats using preclinical brain MRI and whole-body PET/CT. The number of brain metastases and the total brain metastases volume did not significantly differ between rats whether they were injected with MPIO-labeled or unlabeled cancer cells. Also, the number of metastasis affected bones did not significantly differ between the groups indicating that MPIO-labeling did not cause the metastasis development outside the rat brain. Furthermore, tumors had an identical appearance after injection of MPIO-labeled or unlabeled cancer cells, showing that the overall function of cancer cells was also not affected by MPIO-labeling [10].

To our knowledge, bone metastasis development after intracardiac injection with the brain-seeking subclone of the MDA-MB-231 cell line has only been described by Song et al. [28]. Nude rats were inoculated with MDA-MB-231br cancer cells transfected with firefly luciferase, which caused brain metastases and unexpected bone metastases. When a mouse was injected with these cancer cells bone metastasis development was also observed [28]. Song et al. suggested the transfection of the MDA-MB-231 cell line with luciferase as a possible reason for the development of bone metastasis. Here, we made similar observations in our rat model using GFP-transduced MDA-MB-231br cancer cells. However, in contrast to the study of Song et al., we observed almost no bone metastasis development when mice were inoculated with 50.000 MDA-MB-231br/eGFP cancer cells. The average number of metastasis affected bones was almost negligible in mice, while abundant in rats. The occurrence of bone lesions demonstrates that this rat model is currently not suited for investigating brain metastasis and associated therapeutic strategies, as peripheral metastases hamper long-term follow-up. Moreover, our results suggest that the metastatic propensity of the MDA-MB-231br/eGFP cancer cell line outside the brain is species-dependent. This is not surprising as the MDA-MB-231br cell line was isolated in mice by Yoneda et al. [8].

In conclusion, we state that this rat model is currently not suited for investigating brain metastasis as a single disease and testing associated therapeutic strategies because of early and abundant formation of metastases with the MDA-MB-231br/eGFP cell line outside the rat brain, more specific to the bone. Therefore, the metastatic propensity of the cell line uniquely to the brain will be enhanced by species-specific passaging in vivo in rats.

## Supporting information

S1 File The ARRIVE guidelines checklist. Completed ARRIVE Guidelines Checklist for reporting animal data in this manuscript.
(PDF)
